# Genetic Characterization of Porcine Circovirus 3 Strains Circulating in Sardinian Pigs and Wild Boars

**DOI:** 10.3390/pathogens9050344

**Published:** 2020-05-02

**Authors:** Silvia Dei Giudici, Giulia Franzoni, Piero Bonelli, Pier Paolo Angioi, Susanna Zinellu, Viviana Deriu, Tania Carta, Anna Maria Sechi, Francesco Salis, Francesca Balzano, Annalisa Oggiano

**Affiliations:** 1 Department of Animal Health, Istituto Zooprofilattico Sperimentale della Sardegna, 07100 Sassari, Italy; giulia.franzoni@izs-sardegna.it (G.F.); piero.bonelli@izs-sardegna.it (P.B.); pierpaolo.angioi@izs-sardegna.it (P.P.A.); susanna.zinellu@izs-sardegna.it (S.Z.); tania.carta@izs-sardegna.it (T.C.); annamaria.sechi@izs-sardegna.it (A.M.S.); annalisa.oggiano@izs-sardegna.it (A.O.); 2 Department of Veterinary Medicine, Università degli Studi di Sassari, Facoltà di Medicina Veterinaria, Via Vienna 2, 07100 Sassari, Italy; vivideriu@gmail.com; 3 Freelance Veterinary Practitioner, Via Minerva, Ploaghe, 07017 Sassari, Italy; salisfra@tiscali.it; 4 Department of Biomedical Sciences, Università degli Studi di Sassari, Viale San Pietro, 07100 Sassari, Italy; mariafrancesca22@virgilio.it

**Keywords:** PCV3, epidemiology, phylogenesis, pigs, wild boars

## Abstract

Porcine circovirus 3 (PCV3) is a recently discovered member of the Circoviridae family. So far, its presence has been reported in North America, Asia, South America, and Europe. In this study, blood and tissue samples from 189 Sardinian suids (34 domestic pigs, 115 feral free ranging pigs, and 39 wild boars) were used to genetically characterize the PCV3 strains from Sardinia. PCV3 infection in the animals was confirmed by real time PCR. The detection rate in the three groups analyzed was l7.64% in domestic pigs, 77.39% in free ranging pigs, and 61.54% in wild boars. Moreover, our results showed that co-infection of PCV3 with other viruses is quite a common occurrence. Molecular characterization of Sardinian PCV3 strains was performed by sequencing 6 complete genomes and 12 complete cap genes. Our results revealed that there is a high similarity between our strains and those identified in different countries, confirming the genetic stability of PCV3 regardless of geographical origin. Haplotype network analysis revealed the presence of 6 whole genomes or 12 unique ORF2 haplotypes and a nonsynonymous mutation in ORF2 that leads to an R14K amino acid substitution. Phylogenetic analysis of whole genome and ORF2 was also conducted. The Sardinian strains were allocated in three different clusters of phylogenetic trees of both complete genome and ORF2. With this study, we have provided a snapshot of PCV3 circulation in Sardinia. Our findings might help to achieve a deeper understanding of this emerging porcine virus.

## 1. Introduction

Porcine circoviruses (PCV) are small, single-stranded, non-enveloped DNA viruses with a circular genome [1]. Three species in the genus *Circovirus* have been identified: PCV1, PCV2, and PCV3 [2]. Recently, a new circovirus was identified in China and tentatively designated as porcine circovirus type 4 (PCV4) [3]. PCV1 was initially described in the 1970s and it is regarded as non-pathogenic [4], whereas PCV2, first isolated in the early 1990s, has important financial impact on pork production due to increased mortality, reproduction failure, and reduced growth rate in pigs infected with PCV2 [5]. PCV2 infection can be subclinical or associated with several syndromes, commonly known as porcine circovirus–associated diseases (PCVAD) [5,6]. Nowadays, PCV2 is considered endemic in many pork-producing countries.

PCV3 was only recently discovered. In 2016, Palinski et al. reported the detection of PCV3 in North Carolina in sow displaying reproductive problems and clinical signs of porcine dermatitis and nephropathy syndrome (PDNS) [7]. In the same year, Phan et al. described the presence of PCV3 in North America in pigs affected by cardiac and multi-organ inflammation [8]. Since it was first detected in North America, PCV3 has been subsequently detected in Asia, South America, and Europe [9,10,11,12,13,14]. In addition, retrospective studies suggest that this virus has been circulating in domestic pigs and wild boar for decades, and its first detection was traced back to 1966 [9,12,15]. In a recent study, a phylodynamic approach suggested an ancient origin of PCV3 that was backdated to before the 1900s; however, depending on the dataset analyzed, a far more ancient origin was supported, in the order of several centuries or even millennia [16].

PCV3 has been associated with different clinical syndromes, such as abortion and reproductive disorders [7], PDNS, cardiac and multi-systemic inflammation, and respiratory diseases [7,8,17]; nevertheless, it has also been detected in asymptomatic pigs [12,16]. Elements determining whether PCV3 infection would result in asymptomatic disease or fatal outcome are still unknown.

PCV3 possesses a 1999–2001 nucleotide circular genome, with two open reading frames (ORFs), ORF1 and ORF2, encoding for Rep and Cap proteins respectively, and a putative ORF3, which encodes for a putative 231 amino acid (aa) protein of unknown function [8,18]. Phylogenetic analysis suggests a bat origin of this virus and several studies have recognized the cross-species transmission ability of the virus with its genome being detected not only in pig and wild boar samples, but also in samples of dogs, cattle, mice, and even ticks [2]. Different PCV3 classification schemes have been proposed. A subdivision into three groups called PCV3a, PCV3b, and PCV3c, or into two major groups named “PCV3a” and “PCV3b”, has been proposed [9,19,20,21]. A recent study used the largest datasets available of full genome and ORF2 region to analyze the possibility of defining PCV3 genotypes and found only two clades [22]. One of these contains the majority of the known sequences, the other only two distantly related Chinese sequences collected in 2006. So, the authors formally proposed a definition of one PCV3 genotype (PCV3a) [22].

To our knowledge, this is the first study to report the presence of PCV3 in Sardinia. PCV3 infection in domestic pigs, free ranging pigs, and wild boars was investigated. The co-infection of PCV3 and two other porcine viruses, PCV2 and porcine parvovirus (PPV), was also assessed. The full-length genomes of six PCV3 strains and the cap protein gene of 12 PCV3 strains were sequenced and analyzed phylogenetically to determine the genetic characteristics of this emerging virus in Sardinia.

## 2. Results

### 2.1. PCV3 Detection

The presence of PCV3 in Sardinian domestic pigs, free ranging pigs, and wild boars was investigated. As displayed in Table 1, the viral genome was detected in 17.64% (6 out of 34) domestic pigs, 77.39% (89 out of 115) feral free ranging pigs, and 61.54% (24 out of 39) wild boars.

The mean threshold cycle (Ct) values of the positive samples in the three groups that were analyzed were 31.15 ± 7.42, 31.91 ± 4.52, and 29.38 ± 5.43, respectively. Within domestic pigs, the PCV3 genome was detected in three animals (two fattening and one post-weaning pig, Ct 36.49 ± 1.27) and three fetuses (belonging to two different litters, Ct 24.03 ± 5.49). Among the fetal organs of domestic pigs analyzed, the viral genome was detected in three spleens, whereas it was detected in one brain, one blood, and one liver of fattening/post-weaning pigs.

Concerning the type of tissue, the viral genome was detected in the spleen (75%), lung (50%), and blood (66.67%) of PCV3 positive wild boars.

The frequency of PCV3 detection was significantly lower in domestic pigs than in free ranging pigs and wild boars (χ2 = 11.53, p = 0.0007; χ2 = 6.32, p = 0.0019, respectively), whereas the difference in the frequency of PCV3 detection between free ranging pigs and wild boars was not statistically significant (χ2 = 0.60, p = 0.43).

### 2.2. Co-Infection

Co-infection of PCV3 with PCV2 and PPV was investigated using real time PCR. Co-infections were common in both domestic and feral suids: 100% of PCV3+ domestic pig samples (both fetus/placenta and domestic pigs) (6 out of 6), 93.25% of PCV3+ free ranging pigs (83 out of 89), and 95.83% of PCV3+ wild boars (23 out of 24) were co-infected with PCV2 and/or PPV. The frequency of detection of all the three viruses in the different groups analyzed is reported in Table 1.

Within the PCV3 positive samples, the mean Ct values of the PCV2 positive samples were 22.27 ± 6.16 in domestic pigs, 29.42 ± 4.37 in domestic pig aborted fetus/placenta, 26.27 ± 5.94 in free ranging pigs, and 26.26 ± 6.41 in wild boars. Within PCV3 positive samples, the mean Ct values of the PPV positive samples were 37.54 ± 1.85 in domestic pigs, 30.68 ± 5.35 in domestic pig aborted fetus/placenta, 27.21 ± 5.61 in free ranging pigs, and 28.03 ± 5.73 in wild boars.

### 2.3. Sequencing and Genotyping

The characteristics of Sardinian PCV3 strains (6 full genomes + 12 ORF2 sequences) along with the GenBank accession numbers are presented in Table 2.

Sardinian PCV3 full genome strains have a genome size of 2000 nucleotides (nt.) and there is high similarity among them (98.6–99.8%). Additionally, they exhibited a high similarity (98.6–99.7%) with the international reference strains that were analyzed. They represent six unique haplotypes as determined through DNASp5 and POPART analysis (Figure S1).

Sardinian PCV3 ORF2 sequences (645 nt.) have a 97.6–100% similarity among them and a 97.3–99.8% similarity with the international reference strains that were analyzed, and they constitute 14 different haplotypes. In particular, the analysis of the entire ORF2 dataset revealed that the strains PCV3SAR9, PCV3SAR12, PCV3SAR16, and PCV3SAR17 belong to the same haplotype as the sequence MF805724 4332-7_Denmark_2017. PCV3SAR2 belongs to the same haplotype as the sequence MG595741 HU/Szerencs/2017 from Hungary, whereas the strains PCV3SAR13 and PCV3SAR14 can be assigned to the same unique haplotype. PCV3SAR1, PCV3SAR3, PCV3SAR4, PCV3SAR5, PCV3SAR6, PCV3SAR7, PCV3SAR8, PCV3SAR10, PCV3SAR11, PCV3SAR15, and PCV3SAR18 represent unique haplotypes in the ORF2 dataset. The data were confirmed by network analysis (Figure S2).

The amino acid sequences of the Sardinian PCV3 strains identified in this study were compared with those of the reference strain KX778720 [8]. Our analysis revealed the presence of 10 amino acid substitutions in the cap protein and 4 in the rep protein of the Sardinian PCV3 strains (Figures S3 and S4). Compared to the international strains belonging to the datasets analyzed, some Sardinian sequences revealed unique amino acid substitutions. PCV3SAR1 and PCV3SAR2 contained the amino acid substitutions V252I and P78A, respectively, in ORF1, and PCV3SAR4 and PCV3SAR15 contained the amino acid substitutions R14K and A75S, respectively, in ORF2.

**Figure 1:**
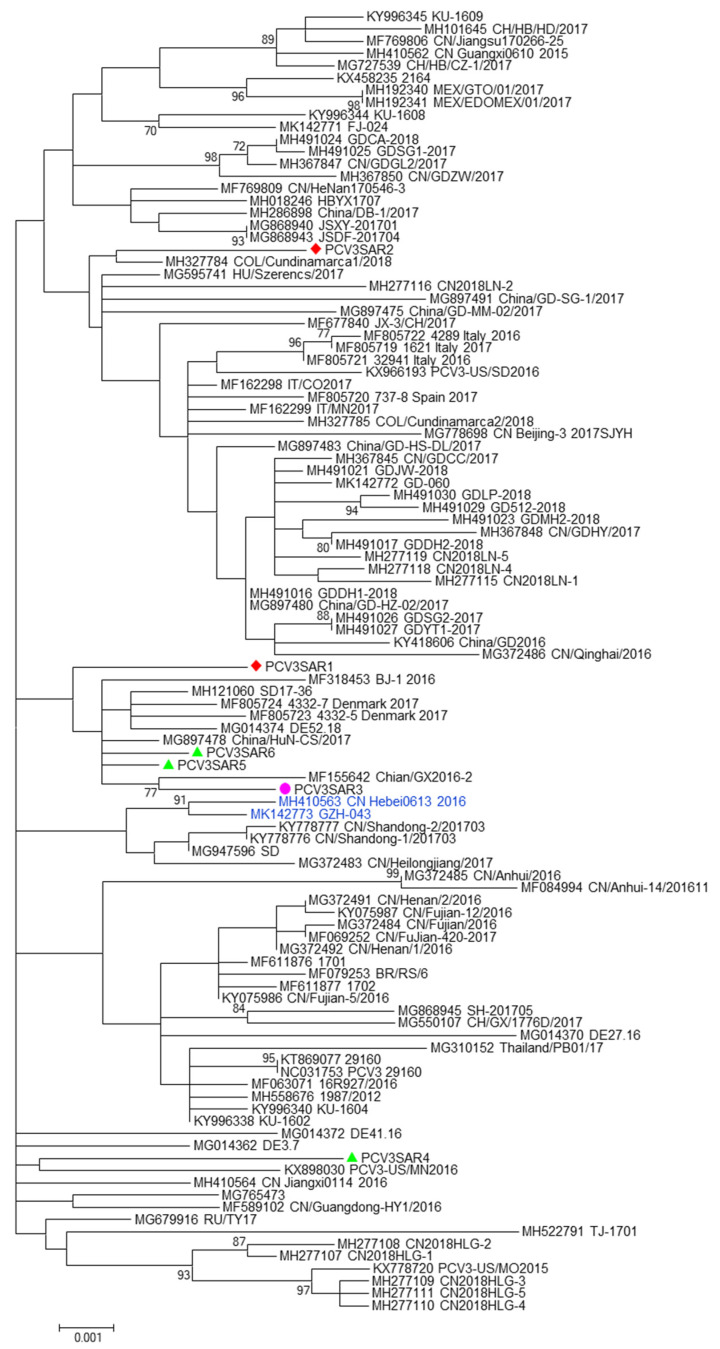
The phylogenetic tree of the PCV3 full genome. The phylogenetic tree was constructed using full genome sequences obtained in this study from different hosts (red: domestic pig; green: free ranging pig; and violet: wild boar) and 100 reference strains retrieved from GenBank. The evolutionary history was inferred using the Maximum Likelihood algorithm with the TrN+I+G method. Evolutionary analyses were performed in MEGA7 software. Bootstrap values < 70 are not shown. Bar: number of substitutions per site.

**Figure 2:**
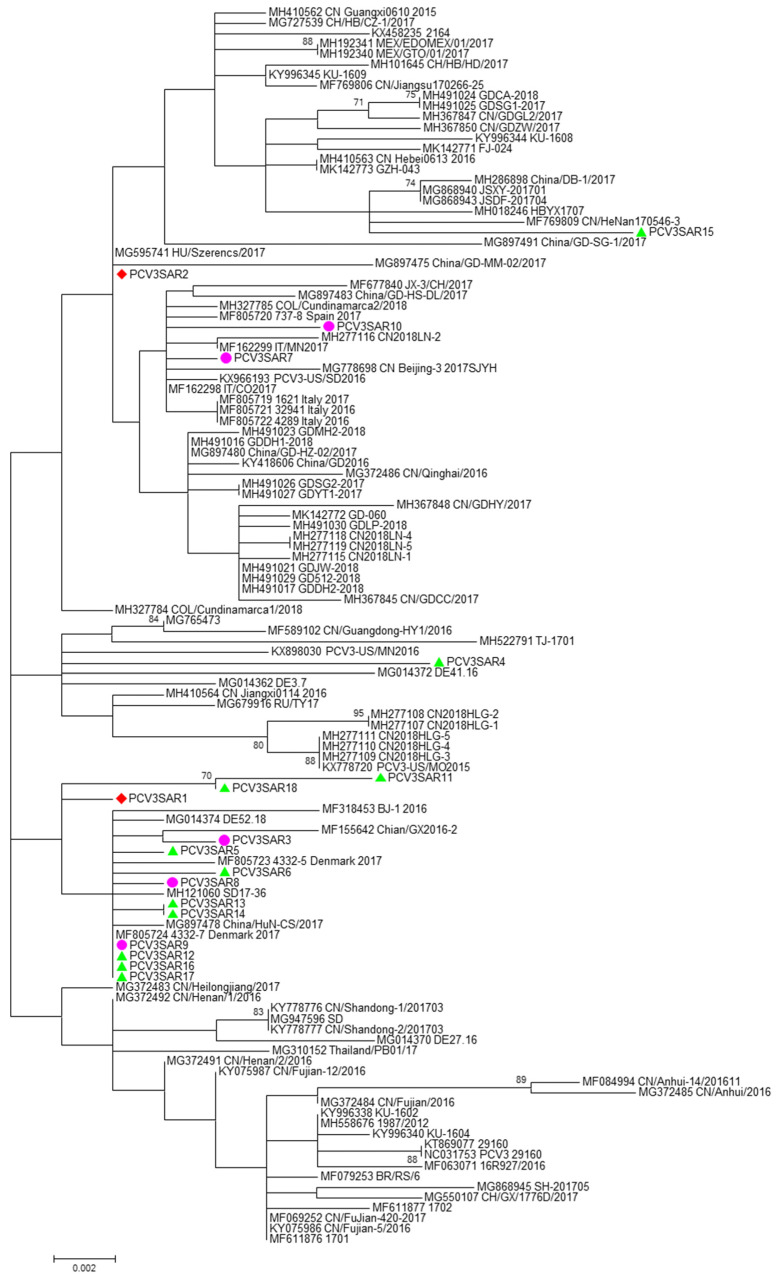
The phylogenetic tree of the ORF2 region of PCV3. The phylogenetic tree was constructed using cap protein gene (ORF2) sequences obtained in this study from different hosts (red: domestic pig; green: free ranging pig; and violet: wild boar) and 100 reference strains retrieved from GenBank. The evolutionary history was inferred using the Maximum Likelihood algorithm with the TrN + G method. Evolutionary analyses were performed in MEGA7 software. Bootstrap values < 70 are not shown. Bar: number of substitutions per site.

No evidence of recombination was detected by RDP3 analysis between the sequences included in the two datasets analyzed. The phylogenetic signal of the datasets, reported in Figure S5, showed the presence of sufficient phylogenetic information. The phylogenetic trees of the complete genome and ORF2 region are reported in Figure 1 and Figure 2, respectively. Sardinian strains belong to the genotype PCV3a recently defined by Franzo et al. [22]. The phylogenetic tree of the full genome revealed that two Sardinian strains clustered with a strain detected in Colombia, Hungary, and China (PCVSAR2), and another strain detected in the USA and Germany (PCV3SAR4). A cluster containing four Sardinian strains (PCVSAR1, PCVSRA3, PCVSAR5, and PCVSAR6), and strains from Denmark, China, and Germany was also found. These results were confirmed by analysis of the ORF2 phylogenetic tree in which 13 out of 18 strains were grouped in the same cluster. The remaining ORF2 sequences clustered with strains from China, Colombia, Spain, Italy, and the USA, confirming the results obtained with haplotype analysis.

## 3. Discussion

This study is the first to describe the presence of PCV3 in Sardinian domestic pigs, free ranging pigs, and wild boars. PCV3 was detected in each of these groups but its detection percentage was significantly lower in samples collected from domestic pigs (20% in domestic pigs, 15.7% in domestic pig fetus/placenta) than in wild boars (77.39%) and free ranging pigs (61.54%). We can speculate that the biosecurity measures adopted in pig farms helped to reduce PCV infection, being effective in reducing environmental contamination by pathogens and minimizing contact between pigs and other animals such as dogs, mice, and ticks, in which PCV3 has been detected [23,24,25]. As already reported by several authors [2,26], PCV3 has also been isolated from wild mammals and hematophagous ectoparasites, thus exhibiting a broad host tropism and wide distribution in wildlife. Accordingly, our data suggests that wild boars might be reasonably considered as reservoirs of PCV3 and, at the same time, free ranging pigs might possibly constitute the link between rural and sylvan environments as already suggested by other authors [27].

Other studies, conducted in Spain, Italy, and Germany, analyzed the circulation of PCV3 in wild boars and reported the detection frequency of 42.66%, 33%, and 23–50%, respectively [26,28,29]. Our results would therefore seem to indicate a greater circulation of PCV3 among the Sardinian wilds compared to other studies. However, we must point out that different PCR methods and tissues were used in these studies.

We detected PCV3 genome in three aborted fetuses, at Ct 24.03 ± 5.49, suggesting its potential role in reproductive disorders. These findings are in accordance with previous studies, where PCV3 was associated with abortion and reproductive disorders in the USA [7,30] and China [31].

PCV3 was identified in sick pigs in the absence of other pathogens and results from a recent study suggest that PCV3 can alone induce PDNS [24]. Besides, co-infections of PCV3 with other swine viruses, such as PCV2, porcine reproductive and respiratory syndrome virus (PRRSV), porcine epidemic diarrhea virus (PEDV), and PPV, are common in pig herds [2,32,33,34,35,36]. In our study, co-infection of two swine viruses (PCV2 and PPV) was assessed and the results showed a large frequency of both the viruses in domestic and free ranging pigs as well as in wild boars. Although further studies are needed to better characterize the PCV3 impact on the pig immune system, we can hypothesize that, by analogy with PCV2 whose immunosuppressive activity has already been demonstrated [37,38], PCV3 infection might compromise the swine immune system to a certain extent, thus facilitating concomitant infection with other common viruses. Indeed, conversely to what we observed, other authors found that even if co-circulation of PCV2 and PCV3 is a common occurrence in pig farms, co-infections of individual pigs seems to be rare [39]. PCV3 nucleotide sequences of 6 complete genomes and 18 complete cap genes, including the 6 from complete genomes, were analyzed in this study. There was high similarity between the Sardinian strains and between Sardinian strains and strains from different countries, confirming that PCV3 is genetically stable regardless of geographical origin [2,19]. This genetic stability was confirmed by the recent determination of the evolutionary rate that was estimated to be in the range of 10^−4^ to 10^−5^ substitutions/site/year using different datasets [16] and all the sequences available at the date of publication.

Six unique haplotypes were found through haplotype analysis of the Sardinian full genome sequences, whereas 12 unique haplotypes out of 14 were recognized through analysis of the ORF2 sequences. The analysis of the ORF2 sequences revealed the presence of unique nonsynonymous mutations in PCV3SAR4, which lead to an R14K amino acid substitution. A recent study provided evidence that the basic amino acid residues at positions 8–32 of ORF2 could direct PCV3 nuclear targeting, and that the highly conserved motif 8RRR-R-RRR16, mutated in PCV3SAR4 and causing replacement of arginine with lysine at position 14, is a key functional nucleolar localization signal that guides PCV3 ORF2 into nucleoli [40]. The function of nucleolar localization signals and that of the amino acid mutation found in PCV3SAR4 needs to be further investigated.

The Sardinian strains were allocated in three different clusters of both complete genome and ORF2 phylogenetic trees (Figure 1; Figure 2). This finding was confirmed by haplotype analysis. All the clusters contained Sardinian strains from the three different pig groups analyzed in the study and from different Sardinian provinces.

Most of the Sardinian strains clustered together, and with strains from Denmark, China, and Germany, as evidenced in both phylogenetic trees and network analysis. These findings suggest that the variability of PCV3 in Sardinia could be due to local viral evolution, and not only due to different epidemic entries. In addition, as we previously speculated in our work on PCV2 [41], we hypothesized that the presence of African swine fever (ASFV) [42], which limits pig trade to imports only, the unique geographical features of Sardinia, and high selective pressure caused by frequent culling of pigs during the ASFV eradication program, together, might have led to generation of new PCV3 variants (not detectable in other countries).

Furthermore, phylogenetic and network analysis (Figure S2) confirmed that our strains clustered with international strains and only two of them clustered with Italian strains. The presence of ASFV in Sardinia prevents the export of live pigs; in the years between 2006 and 2018, pigs were mainly imported from the rest of Italy (30%), and from Spain (24%), France (28%), Germany (9.5%), Belgium (5.8%), and Luxembourg (1.65%) [43]. As opposed to what we already know about PCV2 in Sardinia [41], fewer numbers of Sardinian PCV3 strains clustered with those from other areas of Italy, although in the present study, a lower number of nucleotide sequences were analyzed. To date, only five complete PCV3 sequences are available from the rest of the Italy.

Overall, future studies are needed to better define the characteristics of this recently discovered virus. The reasons behind the broad circulation of PCV3 in the wild are still unknown and comparison between sequences of domestic and wild animals might contribute to a better understanding of the epidemiology of this emerging pathogen and its impact on swine industry.

## 4. Materials and Methods

### 4.1. Samples

In this study, 263 samples were collected between November 2018 and January 2019 and analyzed at the Diagnostic Virology Laboratory of Istituto Zooprofilattico Sperimentale (IZS) of Sardinia:

- 65 samples (aborted fetus, placenta, EDTA blood, spleen, lung, liver, intestine, and brain) from 34 domestic pigs of 16 different farms presenting reproductive failure (8 samples from 6 weaners, 5 samples from 2 post-weaning pigs, 4 from 2 fattening pigs, 5 from 5 sows, and 42 samples from 19 aborted fetus/placenta from 8 litters);

- 83 samples (spleen, lung, EDTA blood) from 39 hunted wild boars in north Sardinia (2018/2019 hunting season);

- 115 spleen samples obtained from feral free ranging pigs culled between 2018 and 2019 in central Sardinia (provinces of Nuoro and Ogliastra) during the depopulation action of the Sardinian ASFV eradication plan.

No clinical data were available for wild boars and free ranging pigs.

### 4.2. DNA Isolation and Detection of PCV3, PCV2, and PPV

Viral DNA was extracted from tissue or blood samples using MagMax Core kit and MagMax96 extractor (Thermo Fisher) following the manufacturer’s instructions; samples were stored at −20 °C until further analysis. All the samples were screened for the presence of PCV3, PCV2, and PPV by qualitative real-time PCR, as previously described [44,45,46]. Samples with Ct value of less than 40 were considered positive.

### 4.3. Sequencing and Phylogenetic Analysis of PCV3 DNA

Six PCV3 positive samples with strong RT-PCR results (Ct values < 30) were fully sequenced. The complete genome sequence of PCV3 was obtained via amplification of three overlapping amplicons produced by PCR using primers previously described [7,11] and reported in Table 3.

Furthermore, the complete sequence of cap protein gene was obtained from 12 positive samples using ORF2 primers described in Table 3. Amplicons were separated using 2% agarose gel and subsequently purified using QIAquick Gel Extraction Kit (QIAGEN), according to the manufacturer’s instructions. Purified samples were stored at −20°C until further analysis.

Sequencing was performed using the primers mentioned before on an ABI-PRISM 3500 Genetic Analyzer (Applied Biosystems, Waltham, MA, USA) and a DNA sequencing kit (dRhodamine Terminator Cycle Sequencing Ready Reaction; Applied Biosystems). The sequences were edited in the BioEdit software version 7.0.0 [47]. ClustalW was utilized for multiple sequence alignment, and BioEdit software and Muscle algorithm of MEGA 7 software were used for editing and sequence translation, respectively [48].

The complete PCV3 genome was assembled and analyzed. Full genome and ORF2 datasets were built using the Sardinian sequences (6 complete strains and 18 ORF2) and all the sequences (n = 100) of international strains retrieved from GenBank in March 2019. Haplotypes were identified using DNASPv5 [49] and POPART software [50]. RDP, GENECONV, MaxChi, and 3Seq methods implemented in the RDP4 software [51] were used to analyze the presence of recombination events. The evolutionary model that best fitted the data of all the datasets was selected using JmodelTest v.2.1.7 [52].

The phylogenetic signal of the datasets was subjected to likelihood mapping analysis with 10,000 random quartets in the TreePuzzle software as already described [41,53]. Phylogenetic trees of the complete PCV3 genome and ORF2 regions were constructed using MEGA 7 software [54] via the maximum likelihood (ML) method and GTR +I +G or TrN +G model of nucleotide substitution, respectively. Statistical support for specific clades was obtained via 1000 bootstrap replicates.

### 4.4. Data Analysis and Statistics

Statistical analyses were carried out using SPSS software v.21. Chi-squared test (χ^2^) was used to analyze the differences in the PCV3 detection rate between domestic pigs, free ranging pigs, and wild boars. P values < 0.05 were regarded as statistically significant.

## Figures and Tables

**Table 1:** Frequency of Porcine circovirus 3 (PCV3), Porcine circovirus 2 (PCV2) and Porcine parvo Virus (PPV) detection.

Host	PCV3 Infection (%)	PCV2 Infection (%)	PPV Infection (%)	PCV3+PCV2 +PPV (%)	PCV3+PCV2 (%)	PCV3+PPV (%)
Domestic pigs	3/15 (20%)	11/15 (57.89%)	2/15 (13.33%)	1/3 (33.33%)	2/3 (33.33%)	0/3
Domestic pig fetuses/placenta	3/19 (15.7%)	16/19 (84.21%)	4/19 (21.05%)	2/3 (66.67%)	1/3 (33.33%)	0/3
Wild boars	24/39 (61.54%)	35/39 (89.74%)	36/39 (92.31%)	23/24 (95.83%)	0/24	1/24 (4.17%)
Free ranging pigs	89/115 (77.39%)	101/115 (87.82%)	85/106 (80.19%)	61/89 (68.5%)	18/89 (20.2%)	4/89 (4.5%)

**Table 2:** Characteristics of Sardinian PCV3 strains. Sequence ID, GenBank accession number, host, year, municipality, and type of sequence obtained are reported.

ID	Accession Number	Host	Year	Municipality (Province)	Sequence
**PCV3SAR1**	MN781187	Domestic pig	2018	Orgosolo (NU)	Full genome
**PCV3SAR2**	MN781188	Domestic pig	2018	Bauladu (OR)	Full genome
**PCV3SAR3**	MN781189	Wild boar	2019	Osilo (SS)	Full genome
**PCV3SAR4**	MN781190	Free ranging pig	2019	Urzulei (OG)	Full genome
**PCV3SAR5**	MN781191	Free ranging pig	2019	Urzulei (OG)	Full genome
**PCV3SAR6**	MN781192	Free ranging pig	2019	Villagrande (NU)	Full genome
**PCV3SAR7**	MN781193	Wild boar	2019	Esporlatu (SS)	ORF2
**PCV3SAR8**	MN781194	Wild boar	2019	Bultei (SS)	ORF2
**PCV3SAR9**	MN781195	Wild boar	2019	Villagrande (NU)	ORF2
**PCV3SAR10**	MN781196	Wild boar	2019	Benetutti (SS)	ORF2
**PCV3SAR11**	MN781197	Free ranging pig	2019	Urzulei (OG)	ORF2
**PCV3SAR12**	MN781198	Free ranging pig	2019	Villagrande (NU)	ORF2
**PCV3SAR13**	MN781199	Free ranging pig	2019	Urzulei (OG)	ORF2
**PCV3SAR14**	MN781200	Free ranging pig	2019	Urzulei (OG)	ORF2
**PCV3SAR15**	MN781201	Free ranging pig	2019	Urzulei (OG)	ORF2
**PCV3SAR16**	MN781202	Free ranging pig	2019	Urzulei (OG)	ORF2
**PCV3SAR17**	MN781203	Free ranging pig	2019	Urzulei (OG)	ORF2
**PCV3SAR18**	MN781204	Free ranging pig	2019	Urzulei (OG)	ORF2

**Table 3:** The details of primers used for sequencing the PCV3 genome.

Primer	Sequence	PCR	Length (bp)	Reference
PCV3_1303F PCV3_8R	5′-ACCGGAGGGGTCAGATTTAT-3′ 5′-TGCCGGGTAATACTAGCC-3′	ORF2	705	[7,11]
PCV3_74F PCV3_927R	5′-CACCGTGTGAGTGGATATAC-3′ 5′-CAAACCCACCCTTAACAG-3′	PCR1	853	[7,11]
PCV3_1303F PCV3_541R	5′-ACCGGAGGGGTCAGATTTAT-3′ 5′-GAGCTGCTGCTTGAAGATCC-3′	PCR2	1238	[11]
PCV3_817F PCV3_1647R	5′-GTTATAATGGGGAGGGTGCT-3′ 5′-GCCTGGACCACAAACACT-3′	PCR3	830	[11]

## Supplementary Materials

The following materials are available online at https://www.mdpi.com/2076-0817/9/5/344/s1. Figure S1: PCV3 whole genome haplotype network, Figure S2: PCV3 ORF2 haplotype network, Figure S3: rep protein comparison between Sardinian PCV3 strains and PCV3-USMO2015 reference strain, Figure S4: cap protein comparison between Sardinian PCV3 strains and PCV3-USMO2015 reference strain, and Figure S5: phylogenetic signal of the datasets analyzed in this study.
