# Supplementary figures and images for: Genetic Characterization of Porcine Circovirus 3 Strains Circulating in Sardinian Pigs and Wild Boars
Source: Pathogens. 2020 May 2;9(5):344. doi: 10.3390/pathogens9050344 (PMC7280999; doi:10.3390/pathogens9050344)

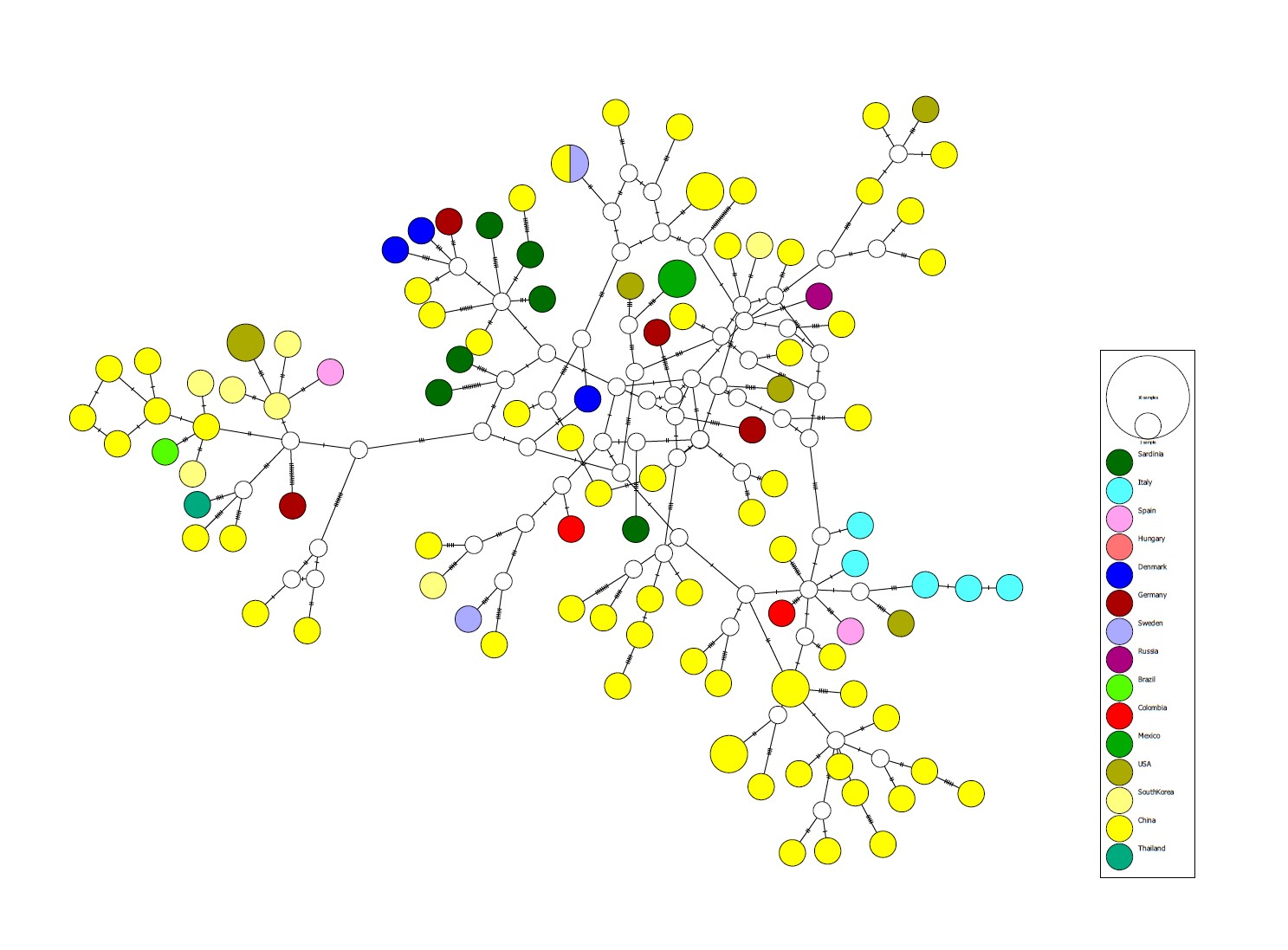

Supplement: Supplementary file 1 [file pathogens-09-00344-s001.zip › supplementary/Figure S1.jpg]

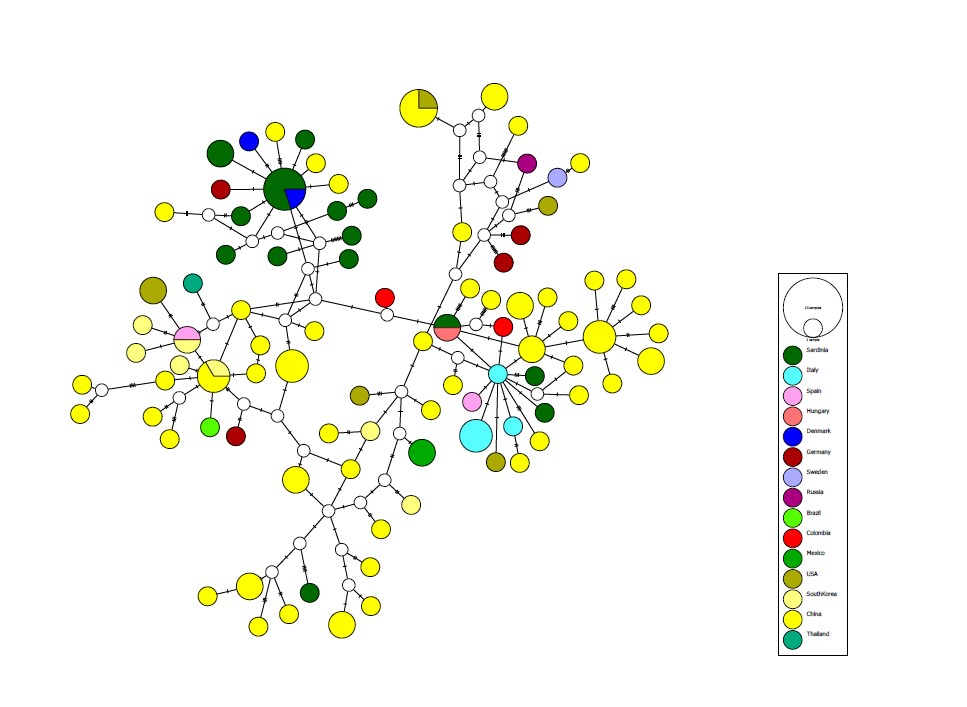

Supplement: Supplementary file 1 [file pathogens-09-00344-s001.zip › supplementary/Figure S2.jpg]

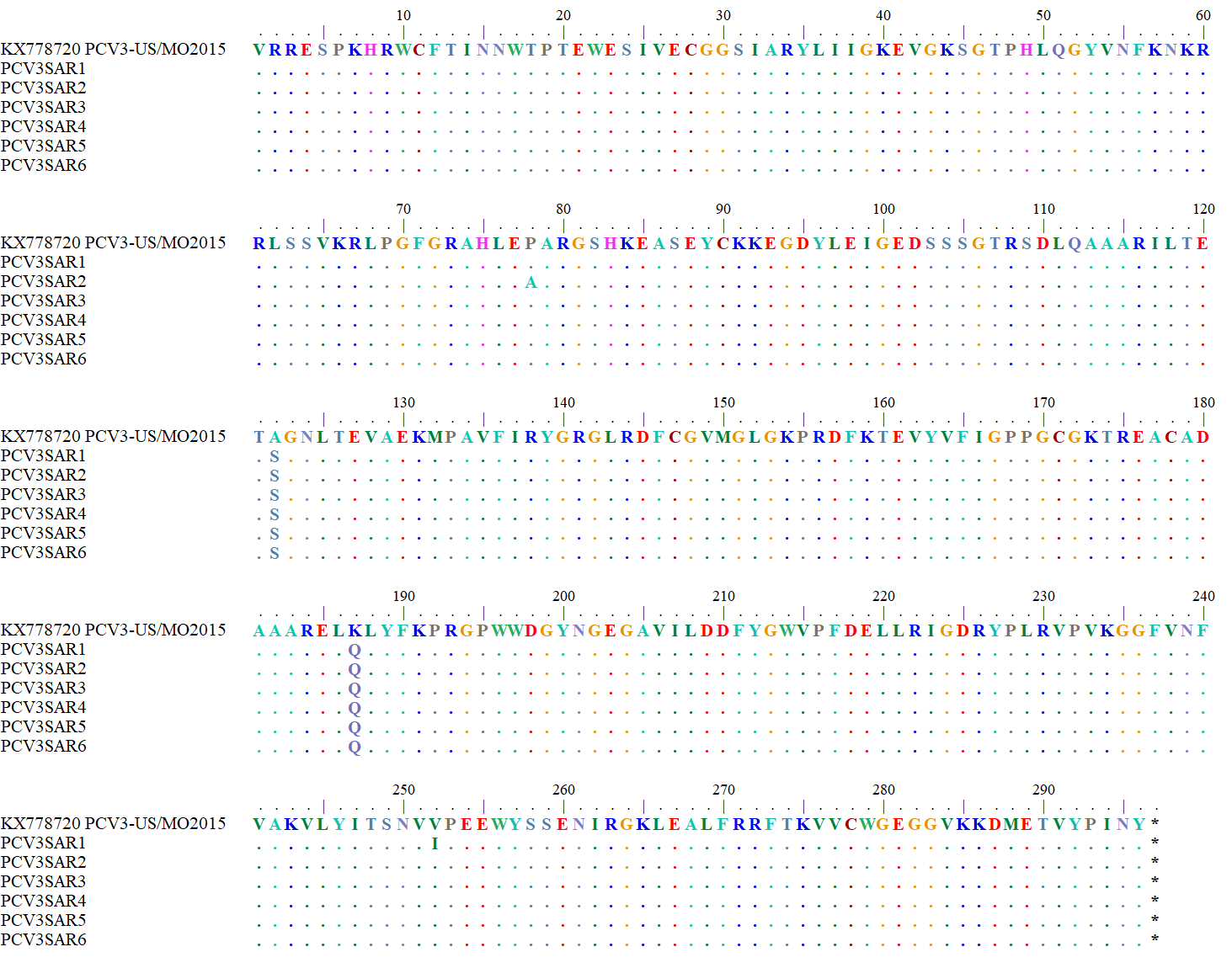


Figure 3. rep protein comparison between Sardinian PCV3 strains and PCV3-USMO2015 reference strain.

Supplement: Supplementary file 1 [file pathogens-09-00344-s001.zip › supplementary/Figure S3.docx]

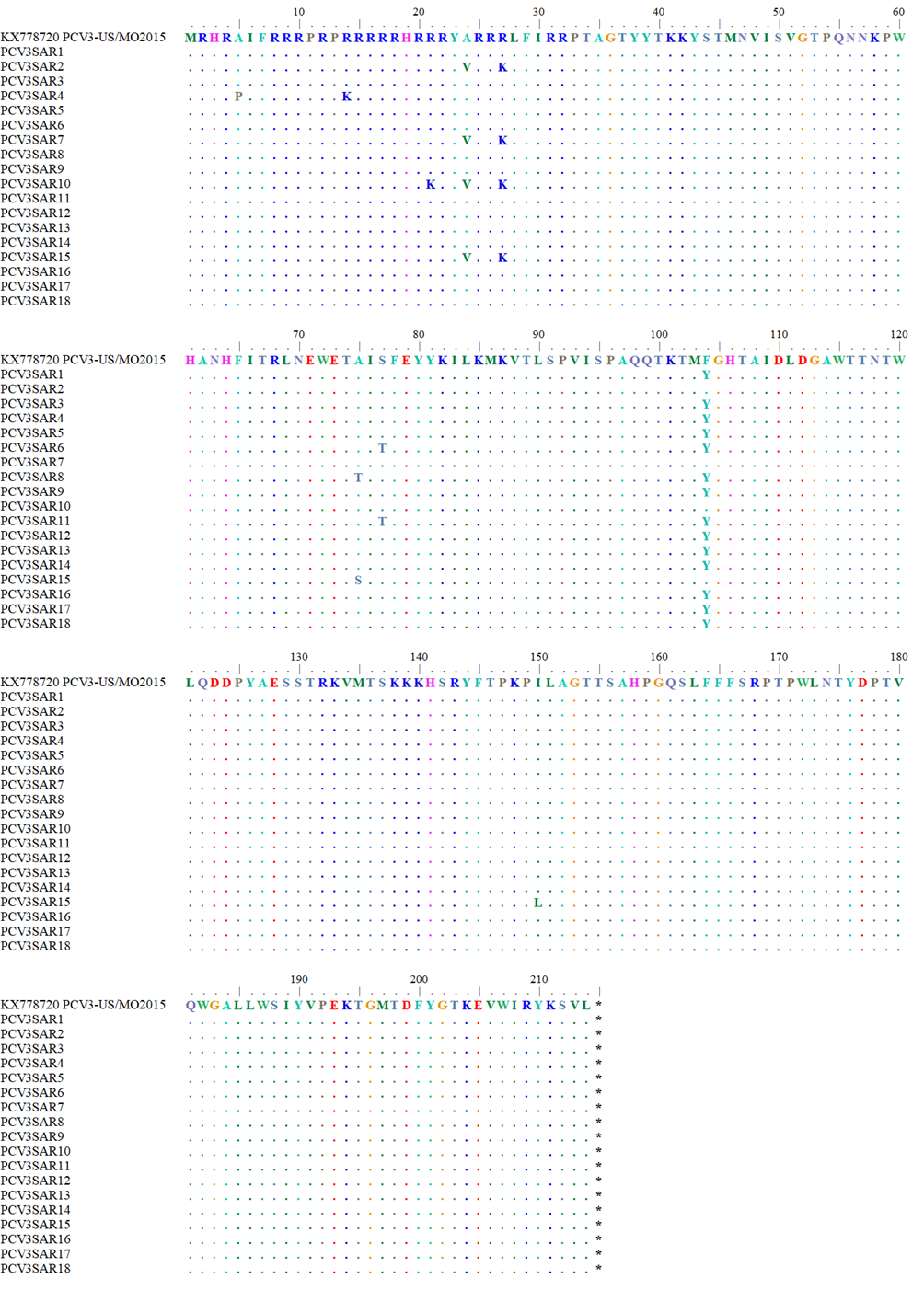


Figure S4: cap protein comparison between Sardinian PCV3 strains and PCV3-USMO2015 reference strain

Supplement: Supplementary file 1 [file pathogens-09-00344-s001.zip › supplementary/Figure S4.docx]

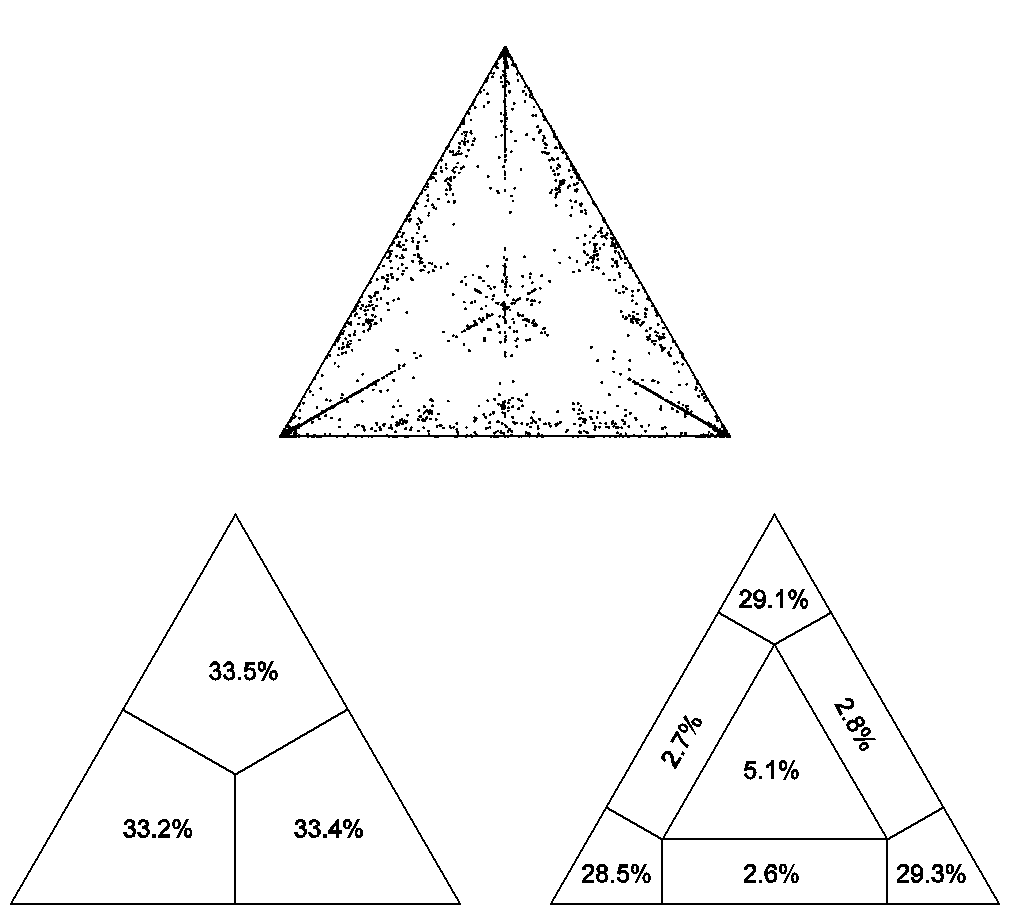

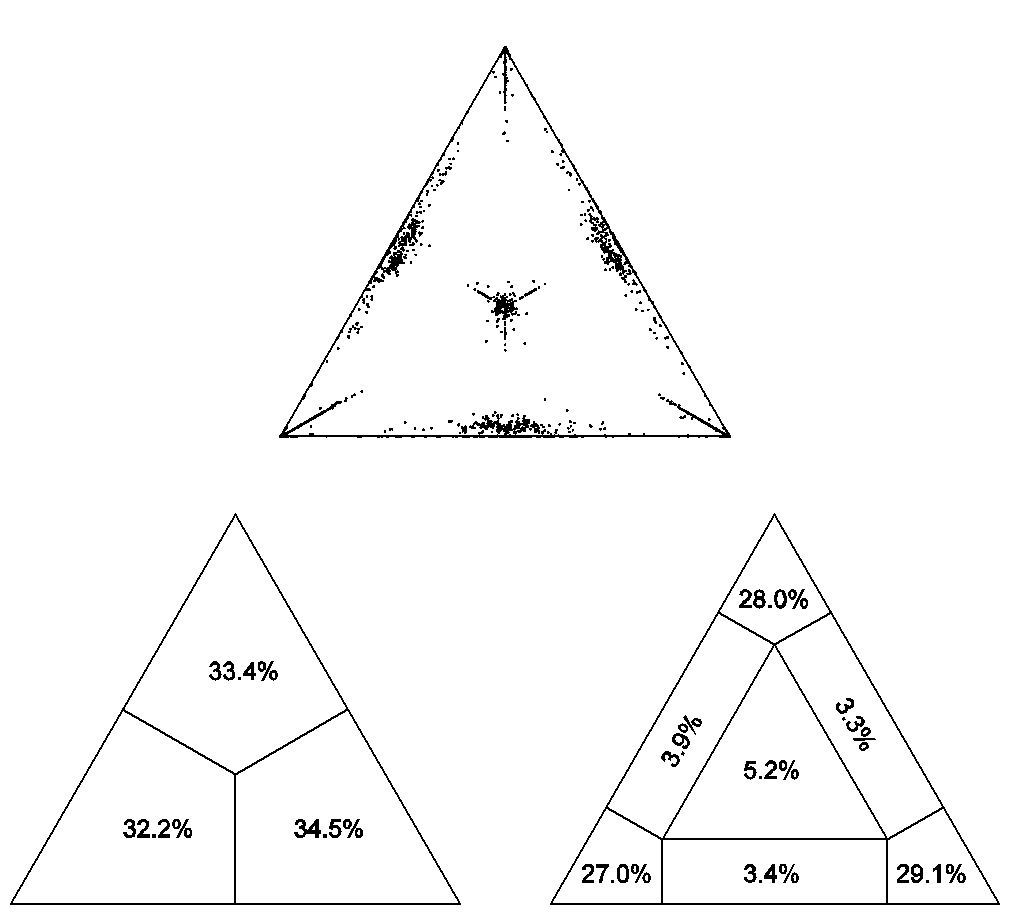


**b**

**a**


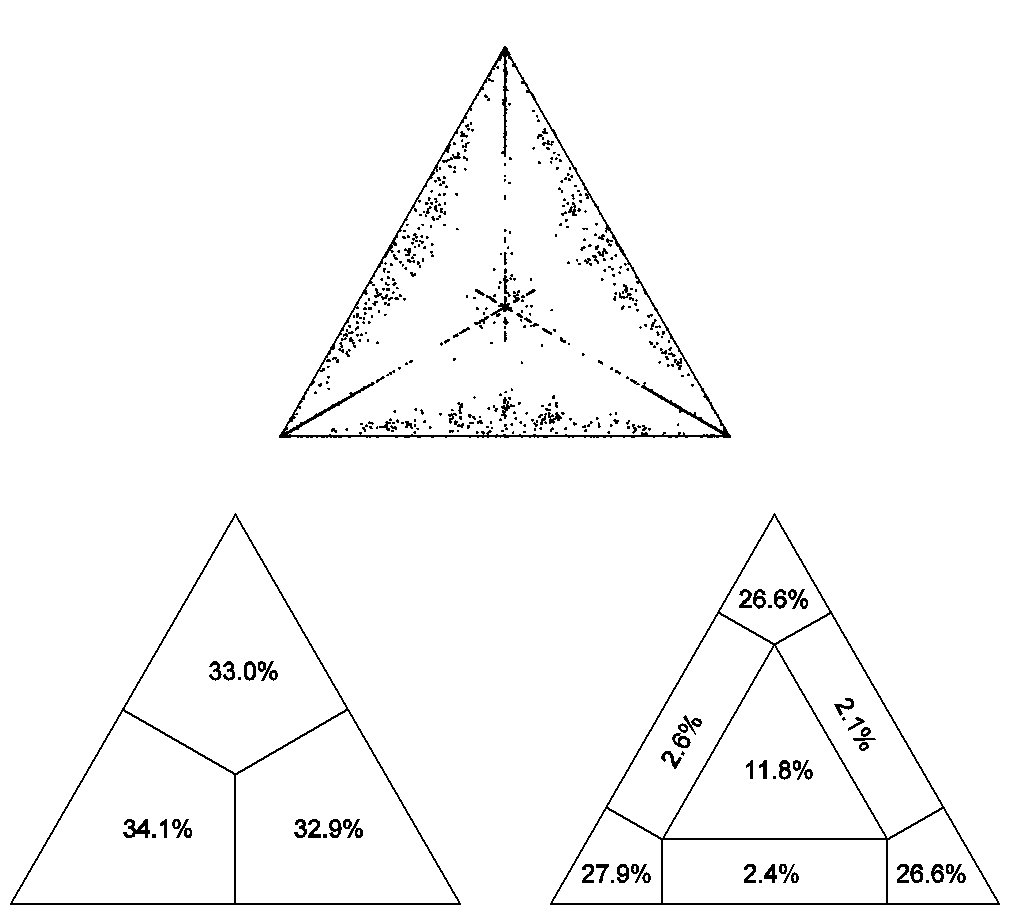


**c**

Figure S5: phylogenetic signal of the datasets analysed in this study.

Supplement: Supplementary file 1 [file pathogens-09-00344-s001.zip › supplementary/Figure S5.docx]
